# Development of InDel markers for *Oryza sativa* ssp. javanica based on whole-genome resequencing

**DOI:** 10.1371/journal.pone.0274418

**Published:** 2022-10-10

**Authors:** Weixiong Long, Yonghui Li, Zhengqing Yuan, Lihua Luo, Laiyang Luo, Weibiao Xu, Yaohui Cai, Hongwei Xie

**Affiliations:** 1 Jiangxi Super-rice Research and Development Center, Jiangxi Academy of Agricultural Sciences, National Engineering Laboratory for Rice, Nanchang, China; 2 State key Laboratory of Hybrid Rice, Wuhan University, Wuhan, China; ICAR - IIRR: Indian Institute of Rice Research, INDIA

## Abstract

*Oryza sativa* ssp. javanica rice varieties exhibit a wide variation in the phenotypes of several important agronomic traits, including grain quality, grain shape, plant architecture, disease resistance, and high adaption to an unfavorable environment, indicating a great potential for rice improvement. DNA molecular markers are basic and critical tools in genetic analysis and gene mining. However, only a few whole-genome variation analyses have been performed in *Oryza sativa* ssp. Javanica (tropical *japonica* rice), and this has hampered the utilization of such an important resource. In this study, the length of insertions/deletions variation greater larger than 10 bp from 10 *Oryza sativa* ssp. indica rice and 10 *Oryza sativa* ssp. tropical japonica rice were extracted by using the Nipponbare genome as a reference. A total of 118 primer pairs which were almost evenly distributed on each chromosome corresponding to the loci of InDels were designed by the Primer 5 program. We confirmed 85 InDel markers from 60 rice varieties, including *indica* and tropical *japonica*, by running polyacrylamide gels. The InDel markers function like SSRs in identifying hybrids, calculating genetic distance, constructing the genetic linkage map, and gene mining. The InDel markers developed in this study might help in genetic studies and to investigate the tropical japonica rice varieties.

## Backgrounds

*Oryza sativa* spp. javanica, also known as tropical *japonica* rice, is famous for its late-maturation, tall stalk, long spikelet, large grains, wide and light green leaves, weak tillering ability, light photosensitivity, and poor shattering. It is widely distributed in Malaysia, Indonesia, the Philippines, and some other regions [1]. Compared to the traits of the *indica* and temperate *japonica* varieties, the *javanica* rice variety exhibits a wide variation in several important agronomic traits, such as high-temperature tolerance, lodging resistance, larger panicles and grains, well-developed root system, and resistance to rice blast. Hence, it might be regarded as a genetic resource for future rice improvement [2–4]. Furthermore, *javanica* rice might be utilized for heterosis and can be a resource for developing highly adaptable hybrid rice.

Breeding varieties with high-yield, high quality and high-combining abilities by hybridizing *indica* and *japonica* has become an important technical approach for utilizing the heterosis of the subspecies [5–7]. However, there are several problems in utilizing heterosis between the two subspecies, which include low seed setting rate, high plant height, low grain fullness, long heading date, and low ecological adaptability [8, 9]. Hybridization between *indica*-*javanica* and *javanica*-*japonica* can overcome the limitations of the *indica-japonica* hybrid [10–12]. Previous studies have shown that the degree of heterosis in rice follows the order: *indica*-*japonica* > *indica*-*javanica* > *japonica*-*javanica* > *indica*-*indica* > *japonica*-*japonica* hybrid. Hence, it is important to understand the genetic variation in *Oryza sativa* ssp. javanica for further utilizing the heterosis of the subspecies.

DNA markers play an important role in genetic analysis and breeding programs, including estimating genetic diversity, construction of the evolutionary tree, population structure analysis, construction of genetic linkage maps, identification of QTLs, gene mining, molecular assisted breeding, and characterization of alien introgression of cultivar rice. To effectively utilize the DNA variation, three generations of DNA markers have been developed, which include restriction fragment length polymorphism (RFLP) by restriction enzyme digestion and hybridization with isotope-labeling [13], rapid amplified polymorphic DNA (RAPD) [14], cleaved amplified polymorphic sequence (CAPS) [15], simple sequence repeats [16], single nucleotide polymorphism (SNP), and insertion/deletion (InDel) markers [17–20]. The first-generation DNA markers (RFLP) have rarely been used in plant genetics in recent years as it is time-consuming and pollutes the environment. The development of second-generation DNA markers such as SSRs are based on a variable number of short tandem repeats and the separation is based on polymerase chain reaction (PCR), followed by agarose or polyacrylamide gel electrophoresis. The characteristics such as codominance, easy readability, and the random and wide distribution of SSR markers have significantly contributed to genetic analysis, gene mapping, and marker assisted selection (MAS) in crops [21]. Owing to the development of re-sequencing technology, the third-generation DNA markers, including SNP and InDel, are applied based on single nucleotide polymorphism (SNP) and insertion/deletion variation in the whole genome [22]. These two kinds of markers are quite popular in plant genetic studies because they exhibit codominance, high density, and easy accessibility. However, genotyping with SNP markers is based on resequencing or SNP arrays, which is rarely conducted in most breeding institutions. Moreover, the validation of such SNP markers relies on restriction enzyme digestion and PCR-based gel electrophoretic separation [23]. Hence, SNP genotyping is widely used commercially since it takes a long time to obtain the data sets. The InDel markers are designed to amplify 150–350 bp DNA sequences and contain insertion/deletion sequences with variations greater than 10 bp that can be easily separated by agarose or polyacrylamide gel electrophoresis due to the presence of a large polymorphic region. A few genome-wide InDel markers have been developed to distinguish the two subspecies in *O*. *sativa* [20, 24–26], but there are no reports on the development of InDel markers for discriminating the *Oryza sativa* ssp. javanica and *Oryza sativa* ssp. indica alleles. Therefore, it is important and urgent to develop a genome-wide InDel marker of *Oryza sativa* ssp. javanica rice.

In this study, we developed polyacrylamide-resolvable InDel markers by re-sequencing the whole genome of 10 *indica* rice varieties and 10 *javanica* rice varieties and identifying DNA sequence variations (insertion and deletion) in the two subspecies by comparing them with the reference genome sequence of the japonica rice variety Nipponbare. The development and characterization of these InDel markers depend greatly on the separation of their PCR products through polyacrylamide gel electrophoresis, determination of their polymorphism and genetic distance, and relationship of the *javanica* varieties. A total of 60 rice varieties, including 30 *indica* and 30 *javanica* varieties, were used to verify the accuracy and applicability of the InDel markers that were developed based on the variation. This study might provide polymorphic markers for *indica* and *javanica* and lay the foundation for research on heterosis between rice subspecies *indica* and *javanica*.

## Materials and methods

### Plant meterials

A total of 80 rice accessions, including 40 *indica* varieties and 40 *javanica* rice varieties, were analyzed. The *indica* rice varieties were collected from China and the *javanica* rice varieties were obtained from the International Rice Research Institute (IRRI). All the germplasms were planted in the Jiangxi Academy of Agricultural Sciences, Nanchang, China for the investigation of agronomic characters. We selected 10 representative accessions of *indica* and 10 *japonica* varieties for resequencing (S1 Table). The remaining 60 rice varieties were used to test the accuracy of the InDel primer pairs that we developed in this study (S2 Table). The 20 varieties resequencing data generated in this study were submitted to the National Genomic Data Center with the BioProject number PRJNA763248.

### Mapping and characterization of InDel variation

The clean data of 20 rice varieties were generated from the raw data after a strict filtering process (Table 1). Then, the clean data of the 20 rice accessions were mapped on the Nipponbare reference genome using the BWA version and a BAM file was produced [27]. The Samtools software was used to sort the BAM file and the Picard software was used to remove the PCR duplicates [28]. The GATK pipeline was performed to detect the InDels for each sample and a vcf file was exported with InDel calls and quality [29]. To understand the function of the InDel variation, the vcf file was annotated using the snpEff software [30].

**Table 1:** The re-sequencing information of 20 rice varieties.

Subspecies	Sequencing ID	Variety Name	Raw Reads	Clean Reads
*Indica*	R01	R752	18145916	18117663
	R02	Hefengzhan	16732881	16707308
	R03	R458	16404329	16380301
	R09	Yuxiangyouzhan	19594960	19564428
	R10	Haodali	20437203	20406924
	R11	XieqingzaoB	18263185	18235592
	R12	R225	17742018	17714419
	R16	Guinongzhan	18355001	18326915
	R17	YuetaiB	20425253	20394151
	R18	Huazhan	18503276	18474866
*Javanica*	R04	Qamuyan	17117976	17093051
	R05	13B	18255101	18224806
	R06	551	17962140	17930382
	R07	13494	23082133	23282528
	R08	Qipaprt 2	22556756	22523437
	R13	Nam mak	23082133	23048803
	R14	Siew khaw	22976506	22939888
	R15	11390	21220932	21190655
	R19	Meqamunin 2	23002937	22970983
	R20	Bugel	22474942	22444383

### Extraction of InDel-flanking sequences and primer designing

To develop the InDel markers, a flanking sequence of 200 nucleotides on both sides of the InDel was extracted as the target sequence based on the Nipponbare reference by using Samtools. The design of the InDel primers was based on the following criteria: i) The primers for the InDel markers must be highly specific and should not match with other loci in the genome. ii) The PCR products of the InDel should be 150–300 bp long so that the polymorphism among the genotypes can be easily identified on a polyacrylamide gel or a 4% agarose gel. Next, the Primer 5 software was used to design the InDel primers [31]. The InDel primers had the following characteristics: the primers were 18–25 bp long, with an optimum length of 21 bp; the melting temperature, optimum temperature, GC content, and the optimum annealing temperature of the primers were 55 to 63°C, 58°C, 40% to 60%, and 48%, respectively. The designed InDel markers that were uniquely aligned to the reference genome were selected as the primers for further analysis.

### PCR amplification

A total of 118 InDel markers, with almost 10 markers evenly distributed on each chromosome, were selected from the primers pairs designed with the custom scripts. A total of 60 rice varieties were used to examine the accuracy of these designed InDel markers. A total volume of 20 μL PCR reaction mix were used, which included approximately 10–100 ng of DNA template, 10 μL of 2 x PCR mix buffer, 1 μL of each primer (10 nM), and 13 μL of ddH_2_O. The PCR reaction was performed as follows: denaturation at 95°C for 5 min, 38 cycles of denaturation at 95°C for 20 s, annealing at 58°C for 20 s, extension at 72°C for 20 s, and a final extension at 25°C for 5 min. PCR amplification was conducted using a Thermal Cycler (Bio-rad).

### Graphical mapping and validation of InDel markers

The chromosomal location of the InDel markers was obtained based on the genome annotation GFF3 files (accessed on 26 August 2021). The PCR products (1–3 μL) were used for electrophoresis on a 6% polyacrylamide gel, visualized, and recorded as a binary code (0/1) by silver staining using a gel imaging system (Yuejin Machinery Co. Ltd.). Only the InDel markers can easily distinguish the *indica* and *javanica* varieties were selected for further analysis.

## Results

### Identification and distribution of genome-wide InDels

**Fig 1:**
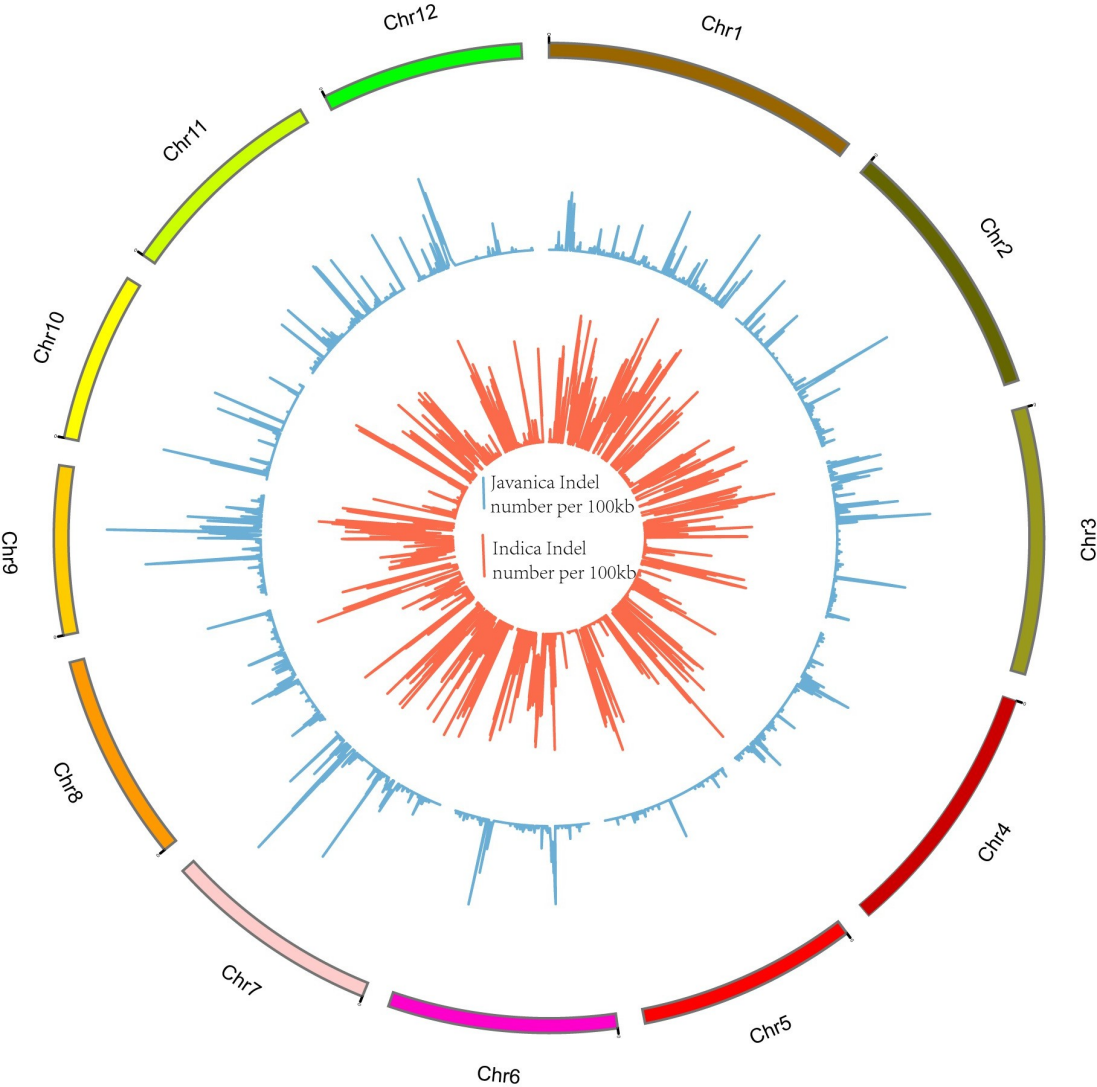
Distribution of InDels in *Oryza sativa* ssp. indica and *Oryza sativa* spp. javanica on each chromosome with a window size of 100 kb. The outermost circle represents 12 rice chromosomes; the middle circle represents the distribution of common InDels in the 10 *indica* rice varieties with Nipponbare as a reference; the innermost circle indicates the distribution of 10 *javanica* common InDels on each chromosome with a window size of 100 kb, orange line indicates the *indica* common InDel number per 100kb, cyan-blue line suggests the *javanica* common InDel number per 100kb.

**Fig 2:**
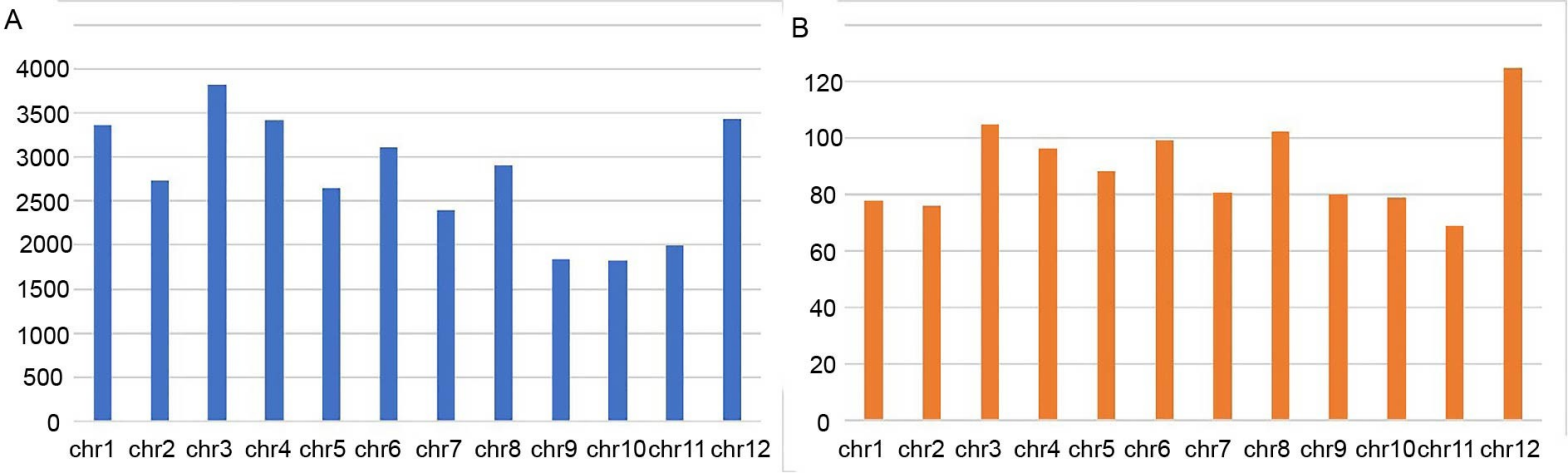
The number and density of InDels in *javanica* rice varieties. A: the common InDels identified on each chromosome from the 10 *Oryza sativa* ssp. javanica sequences with Nipponbare as a reference. B: the density of the InDels (n/Mb) of the 10 *Oryza sativa* ssp. javanica accessions.

High-quality clean reads of 368,645,134 and 423,297,832 were generated for the subspecies varieties of *indica* and tropical *japonica*, respectively (Table 1). A total of 372,946,555 reads for the *indica* group and 393,523,062 reads for the tropical *japonica* group were mapped at an average depth of 10 to the *japonica* Nipponbare reference genome. A total of 115,576 InDels and 33,456 InDels shared by the selected *indica* varieties and tropical *japonica* accessions, respectively, were identified (Fig 1). The number of InDels in tropical japonica on each chromosome ranged from 1,830 (Chr10) to 34,821 (Chr12), with the density ranging from 68.78 InDels/Mb (Chr11) to 104.93 InDels/Mb (Chr12) (Fig 2, S3 Table).

Based on the vcf file, an average of 5,613 InDels on each chromosome were generated by comparing the genotype of the *indica* group with the tropical *japonica* group (S4 Table). Additionally, the distribution of the InDel markers in the entire genome of the two groups of subspecies was also analyzed, the results indicated that the length of the identified InDels on each chromosome was approximately consistent with the length of the corresponding chromosome, except for those on Chr5 and Chr9. The highest number of InDels was mapped on chromosome 1 (12,625), while chromosome 10 exhibited the lowest number of InDels at 1,656. The average density of the InDels on the chromosomes of the two subspecies ranged from 79.36 InDels/Mb on chromosome 10 to 292.19 InDels/Mb on chromosome 9, with an average density of 176.37 InDels/Mb (S4 Table).

### Development of InDel primers

To establish a high-quality InDel marker resource for discriminating the alleles between the *indica* and tropical *japonica* subspecies, certain precautions were taken while designing the InDel primers, which included (i) duplicated/repeat regions in the rice genome were avoided, (ii) InDels that were evenly distributed throughout the genome were selected, and (iii) the alleles could be separated through polyacrylamide gel electrophoresis. To improve the design efficiency of the InDel primers for the tropical *japonica* varieties, we retained the InDels that were shared by the tropical *japonica* varieties but absent in the *indica* group. The location of the InDel polymorphic between one subspecies and the reference genome was established, and the different genotypes of the two subspecies, irrespective of the genotype of the reference genome, were regarded as subspecies-specific InDels. Hence, the 200 tropical *japonica*-specific InDels distributed across the 12 chromosomes were selected for further development of PCR-based markers. The target sequence length of 400 nucleotides, which contained the corresponding insertion/deletion sites, were extracted as templates for primer designing. Considering the separation ability of electrophoresis, the InDels larger than 10 bp were further selected for primer designing. Then, Primer 5 was used to design PCR primers based on several parameters. A total of 118 InDel primers were successfully designed in this study. The name, location, variation, product length, and annealing temperature of the primers are listed in S5 Table.

### Validation of the newly developed specific InDel markers

A set of 118 InDel markers that were almost evenly distributed on the 12 chromosomes were selected, and primers were designed for experimental validation (S5 Table). To determine the accuracy of the designed InDel markers by PCR, we amplified the genomic DNA of 20 samples selected from the two subspecies. Among these primers, 16 pairs were inefficiently amplified or did not include the target sequence from all the resequencing varieties, and hence, were not used for further analysis. Furthermore, 10 of these primers were found to lack polymorphism, with the same production length found in the two subspecies varieties. Seven pairs of primers showed polymorphism, which could not be used to distinguish the *indica* and tropical *japonica* rice accessions. The remaining 85 InDel primers can efficiently distinguished the subspecies varieties and served as markers for tropical *japonica* subspecies in the genetic analysis. To quickly refer to the tropical *japonica* InDel primers, we renamed them as “IJ” (derived from “*Indica*” and “*Javanica*”) and associated it with a number based on the location of the InDel on the chromosome (S6 Table). The InDel primers tightly linked function genes were also listed in S6 Table.

### Application of InDel markers

**Fig 3:**
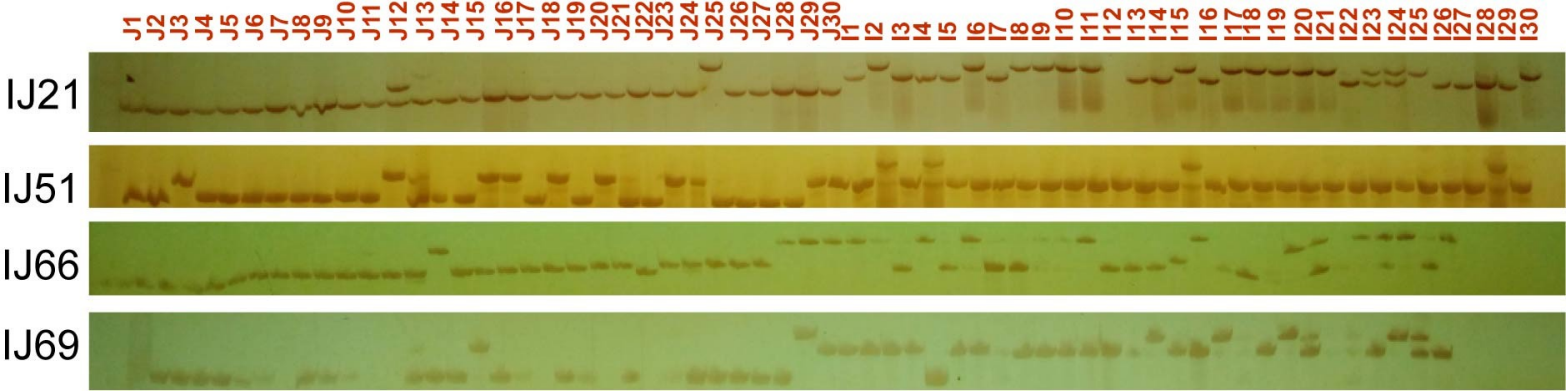
Four representative InDel markers show more than two alleles but can distinguish the two subspecies. The 30 samples on the left and 30 samples on the right show the *Oryza sativa* ssp. javanica varieties and *Oryza sativa* ssp. indica accessions, respectively. The detailed information is presented in S2 Table.

**Fig 4:**
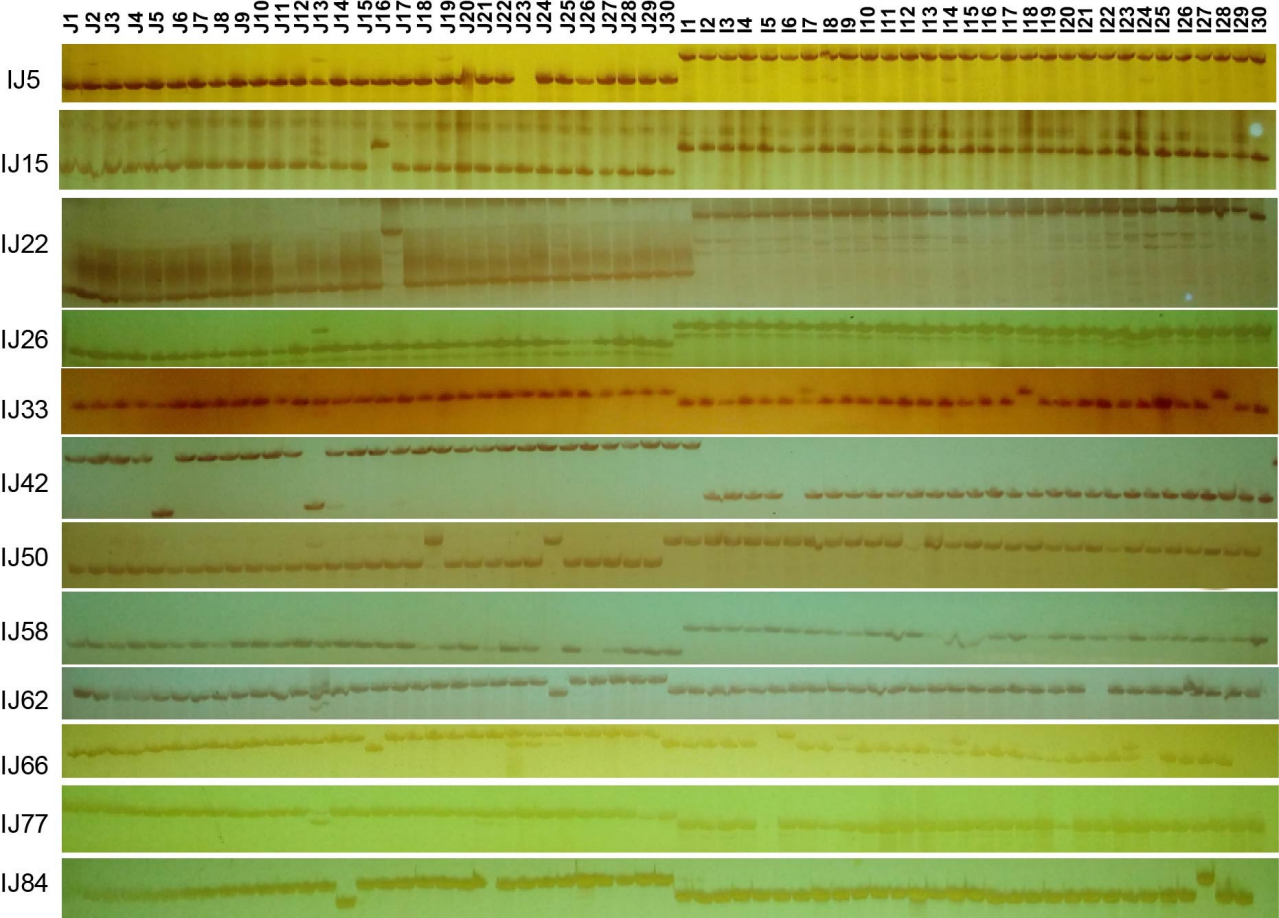
Application of the newly developed InDel markers in 60 rice varieties, including each 30 *indica* and *javanica* accessions. The 30 samples on the left (*javanica*) exhibited single bands different from the 30 samples on the right (*indica*).

**Fig 5:**
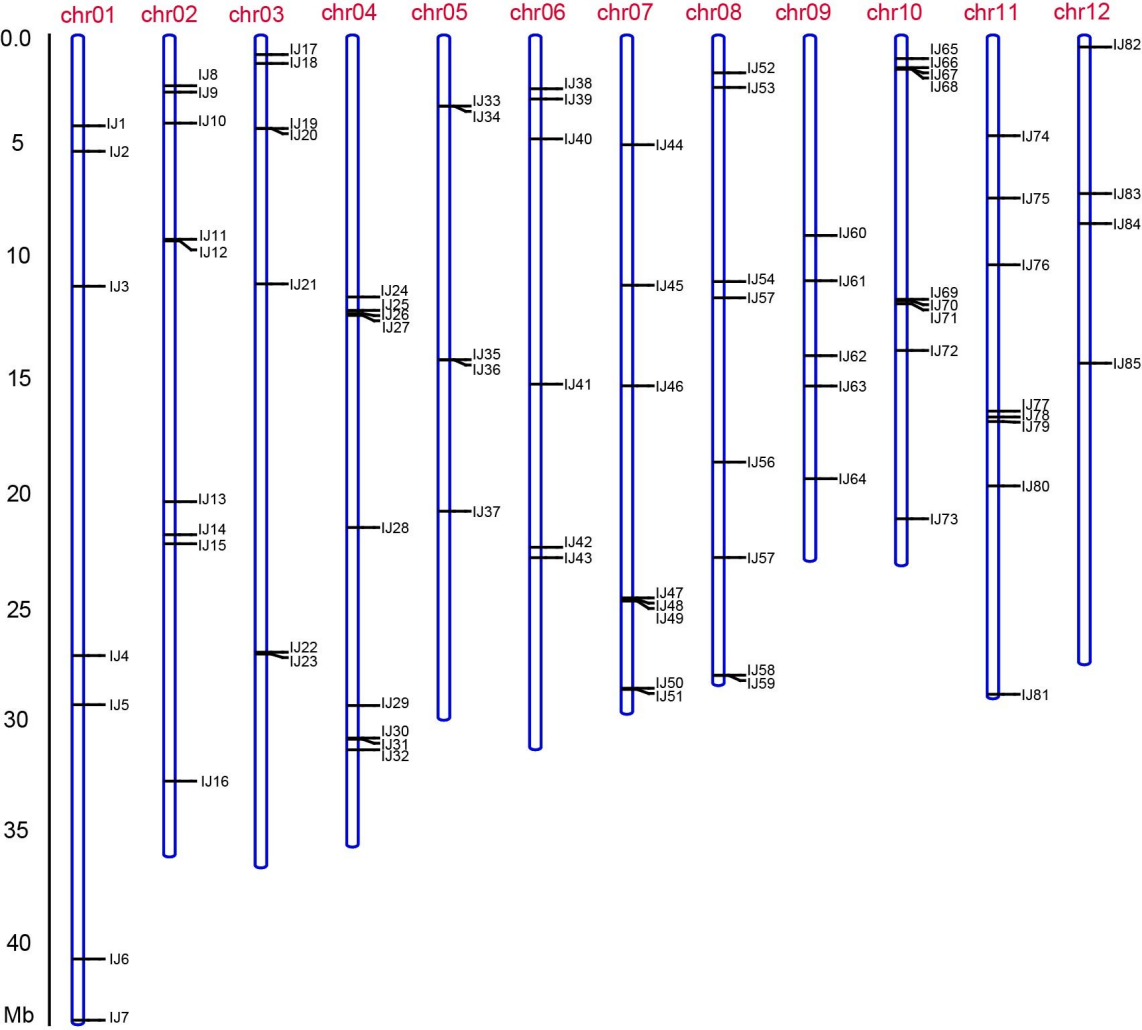
The physical map of 85 available InDel markers. The position of each marker is indicated on the reference genome (Nipponbare-IRGSP-1.0) with a horizontal bar.

In addition to the resequenced rice varieties, 60 rice accessions, including 30 *indica* and 30 tropical *japonica* varieties, were used to investigate polymorphism by using 85 tropical *japonica*-specific InDel primers. Among them, 13 InDel primers with more than two alleles could distinguish the tropical *japonica* group from the *indica* group and a few *indica* varieties harbored the tropical *japonica* alleles (Fig 3). Using polyacrylamide gel electrophoresis, 72 InDel primer pairs with two alleles were found to distinguish the two subspecies (Fig 4). The validation results showed that 85 (100%) InDels could distinguish the tropical *japonica* from the *indica* subspecies. We found that 84.52% of InDels were subspecies specific and could serve as an important source of marker for identifying the tropical *japonica* varieties and calculating the genetic map or genetic distance for developing a new population by hybridization between *indica* and tropical *japonica*. Additionally, chromosome 12 exhibited the lowest number of tropical *japonica-*specific InDel markers, while the highest number of tropical *japonica-*specific InDel markers was located on chromosome 4 (Fig 5).

## Discussion

Rice is a staple food for over 10 billion people, and increases in crop production is crucial to meet the demands of the growing global population. Climate change has threatened food security by reducing crop productivity. Geneticists and breeders are investigating wild rice and landraces for a solution. Tropical *japonica* preserved by local people has already provided the possibility for rice improvement and is recognized as a “genetic reservoir” for further improvement of elite rice varieties [32]. The rice germplasm of tropical *japonica* is highly valuable as it harbors superior alleles, rare alleles, and novel genes compared to that of the cultivated varieties [33, 34]. To utilize tropical *japonica* rice, the genome-wide DNA variation between tropical *japonica* and *indica* should be well-understood foremostly. However, previous studies on DNA polymorphism concentrated on the *indica*, *japonica*, and wild rice varieties [26, 35]. Based on the published reference sequences of *indica* (9311) and *japonica* (Nipponbare), 479,406 InDel regions were identified by comparing the sequences of the two genomes. However, only 108 InDel markers were available for further genetic analysis [20]. By using 1,767 rice resequencing data, 100 high polymorphic InDels were experimentally validated from the 2,329,544 identified regions [24]. A total of 133 polymorphic InDel markers, validated from 506 InDel markers developed based on published sequences of the rice genome, were used to construct the genetic map of *indica* and *japonica* [36]. Based on two *indica* rice genomes and one *japonica* rice genome, 19,937 large InDel markers (30–55 bp) were extracted and 346 markers exhibited polymorphism from 22 cultivars [25]. Additionally, a few sets of genome-wide markers were developed in wild rice. A total of 94 InDel markers for *O*. *officinalis* were developed based on the bacterial artificial chromosome (BAC) end sequences. Twenty-two InDel markers were developed to discriminate all genotypes in the genus *Oryza* by using 12 *Oryza* species, which included 102 wild rice accessions [37]. Recently, 541 InDel markers were developed to discriminate the cultivars and AA-genome wild rice by positional multiple sequence alignments among five AA-genome species with four rice varieties [26]. Our study was the first time to provide tropical *japonica*-specific primers for breeding by investigating genome-wide InDels compared to *indica* with Nippobare as the reference.

Previously, InDels were developed to discriminate between subspecies or species mainly based on two varieties. To accurately determine the polymorphism between the subspecies of *indica* and tropical *japonica*, 20 varieties, including 10 *indica* with polymorphic phenotypes and 10 *javanica* with a wide global distribution, were selected for resequencing in this study. To resolve the PCR products on polyacrylamide gels, only those InDel variations that were larger than 10 bp and PCR products that were 100–300 bp were selected, which reduced the number of available InDels. Ten InDel primers from each chromosome were selected to investigate the polymorphism in another 60 varieties, including *indica* and tropical *japonica*. Seven of these varieties had more than two alleles but the alleles could not be used to discriminate the two subspecies due to the presence of some repeat sequences in both subspecies. Thirteen of them exhibited more than two alleles that could discriminate the two subspecies and probably resulted in heterozygosity. A few samples of *indica* showed two alleles in the 85 tropical *japonica*-specific InDel markers. These results indicated that the *indica* show more sequence duplication or gene family expansion than the corresponding region to the InDel marker production in *javanica* group.

In this study, we obtained 92 polymorphic primers for the subspecies of *indica* and tropical *japonica*. Using 85 of the 92 markers, we developed markers that could discriminate the *indica* and tropical *japonica* varieties. As we can see in the S6 Table, many grain shape related genes was overlapped with our development InDel markers. For example, a QTL for grain length, OsLG3, which encodes MADS-box transcription factor 1 (OsMADS1) were mapped from Oryza *javanica* [38]. Haplotypes and introgression regions revealed that the long-grain allele of *OsLG3b* might have arisen after domestication of tropical *japonica* and spread to subspecies *indica* or temperate *japonica* by natural crossing and artificial selection. Grain shape related gene *TGW2* was identified through Fst and selective sweep analysis, which suggested the differentiation of *TGW2* may contributed to the grain width polymorphism between *javanica* and *indica* subspecies [39]. Furthermore, *GL7*/*GW7* encodes a protein homologous to *Arabidopsis thaliana* LONGIFOLIA proteins, which regulated longitudinal cell elongation was identified from *Oryza javanica* by using a genome-wide association analysis [40]. Interestingly, a tandem duplication of 17.1-kb segment at the GL7 locus leads to upregulation of GL7 and downregulation of its nearby negative regulator. Different natural and artificial selection during the evolution history resulted in the structural variation, including copy number variation, presence and absence variation, and inversion between *javanica* and *inidca* subspecies. These return to give opportunity for us to exploit the huge diversity to improve rice breeding.

To obtain the QTL/gene from Oryza *javanica*, segregated population from hybrids of *javanica* subspecies and other cultivar rice subspecies should be constructed. The labor-saving and economic-saving method to construct the population depending on the efficiency of distinguishing the true hybrids. What’s more, these InDel markers can easily be utilized by most of rice breeders with general equipment. The genome-wide InDel markers in both *indica* and tropical *japonica* in this study can facilitate marker-assisted breeding and functional gene mining.

## Supporting information

S1 Table The information of the 60 tested varieties which including 30 *Oryza sativa* ssp. Indica and 30 *Oryza sativa* ssp. Javanica. This file is in the tab delimited format and can be open using the software Excel.
(XLSX)

S2 Table The information of the 20 sequenced varieties which including 10 *Oryza sativa* ssp. indica and 10 *Oryza sativa* ssp. javanica. This file is in the tab delimited format and can be open using the software Excel.
(XLSX)

S3 Table The total InDels number and InDels density (number per Mb) on each chromosome of the two subspecies. This file is in the tab delimited format and can be open using the software Excel.
(XLSX)

S4 Table The group common Indels number and Indels density (number per Mb) on each chromosome within the two subspecies. This file is in the tab delimited format and can be open using the software Excel.
(XLSX)

S5 Table The detailed information of primers used in this study. This file is in the tab delimited format and can be open using the software Excel.
(XLSX)

S6 Table The renames, location, corresponding to designed primers number, and the tightly linked function genes of the 85 available markers. (XLSX)
